# Adapting the Care Partner Hospital Assessment Tool for Dementia Care

**DOI:** 10.1002/alz.085916

**Published:** 2025-01-09

**Authors:** Beth Fields

**Affiliations:** ^1^ University of Wisconsin‐Madison, Madison, WI USA

## Abstract

**Background:**

Care partners commonly support patients living with dementia (PLWD) during and post‐hospitalization, however, they frequently report feeling excluded from care and unprepared for their caregiving tasks. While the evidence‐informed and validated Care Partner Hospital Assessment Tool (CHAT) was designed to better include and prepare family members and friends, it has not yet been adapted for the growing number of care partners supporting PLWD.

**Objective:**

To adapt and finalize the CHAT for care partners of hospitalized PLWD.

**Setting:**

Hospitals in the United States.

**Study Population:**

We selected hospital‐based clinicians who had at least 5 years of professional experience working with PLWD and their family/friends, and care partners who were at least 18 years old and provided support to a PLWD who experienced a hospitalization. A total of 7 clinicians and 5 care partners were recruited. Clinicians were White (100%), female (85%), and represented social work, nursing, occupational therapy, medicine, and pharmacy. Care partners ranged in age from 41 to 73 years, were White (100%), female (100%), and provided support to a mother or mother‐in‐law (30%), spouse/partner (20%), and other relative (50%).

**Methods:**

Two design teams each consisting of clinicians and care partners respectively—were formed to suggest adaptations to CHAT and share satisfaction with the final tool. Teams completed 5 co‐design videoconference sessions, with 2‐3 weeks between each session. Sessions lasted approximately 60 minutes and were audio recorded. The Rapid Identification of Themes from Audio‐recordings method was used to identify suggestions for adaptation between sessions, and thematic analysis was used to code the transcripts in NVivo.

**Results:**

Adaptations to the CHAT included (a) providing options for dementia specific educational resources, (b) identifying power of attorney and care partner roles and responsibilities (c) using more comprehensive response options and simple language. After making adaptations, participants (100%) reported that they were satisfied or very satisfied with the final Dementia CHAT (i.e., D‐CHAT). Participants described the D‐CHAT as ‘empowering’, ‘comprehensive’, ‘collaborative’, and ‘helpful’.

**Conclusion:**

The converged‐upon D‐CHAT is ready for feasibility testing in the hospital setting and may help guide clinicians’ inclusion and preparation of care partners of PLWD.

